# High-Throughput Mass Spectrometry Imaging with Dynamic Sparse Sampling

**DOI:** 10.1021/acsmeasuresciau.2c00031

**Published:** 2022-08-15

**Authors:** Hang Hu, David Helminiak, Manxi Yang, Daisy Unsihuay, Ryan T. Hilger, Dong Hye Ye, Julia Laskin

**Affiliations:** † Department of Chemistry, Purdue University, West Lafayette, Indiana 47907, United States; ‡ Electrical and Computer Engineering, Marquette University, Milwaukee, Wisconsin 53233, United States

**Keywords:** mass spectrometry imaging, high-throughput imaging, dynamic sparse sampling, deep learning, nanospray desorption electrospray ionization, data-driven experiments

## Abstract

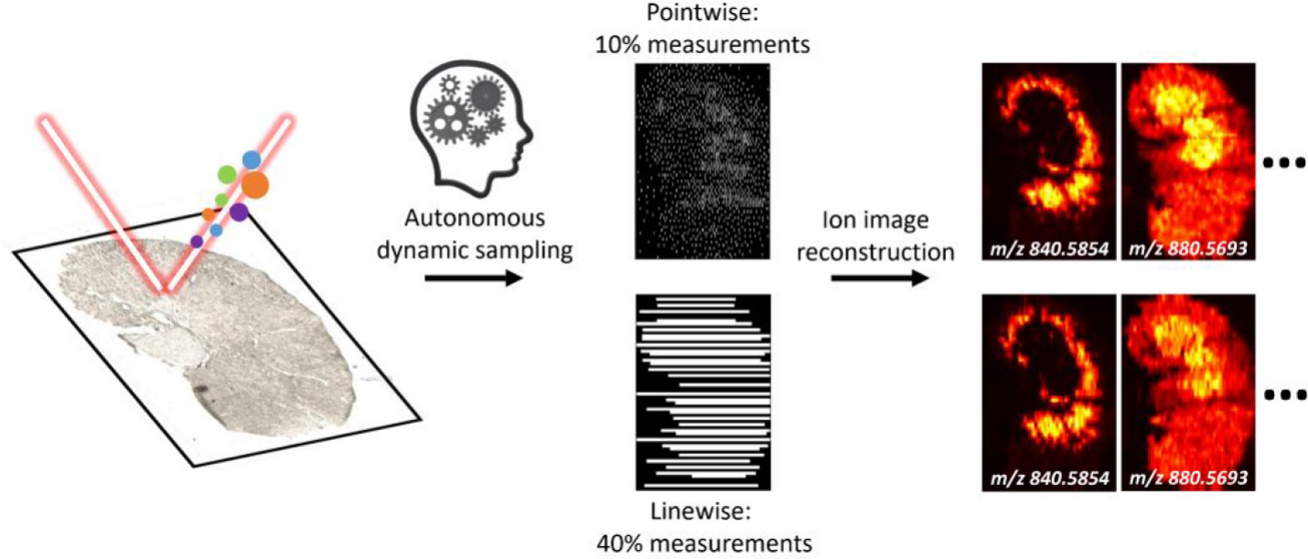

Mass spectrometry imaging (MSI) enables label-free mapping of hundreds of molecules in biological samples with high sensitivity and unprecedented specificity. Conventional MSI experiments are relatively slow, limiting their utility for applications requiring rapid data acquisition, such as intraoperative tissue analysis or 3D imaging. Recent advances in MSI technology focus on improving the spatial resolution and molecular coverage, further increasing the acquisition time. Herein, a deep learning approach for dynamic sampling (DLADS) was employed to reduce the number of required measurements, thereby improving the throughput of MSI experiments in comparison with conventional methods. DLADS trains a deep learning model to dynamically predict molecularly informative tissue locations for active mass spectra sampling and reconstructs high-fidelity molecular images using only the sparsely sampled information. Experimental hardware and software integration of DLADS with nanospray desorption electrospray ionization (nano-DESI) MSI is reported for the first time, which demonstrates a 2.3-fold improvement in throughput for a linewise acquisition mode. Meanwhile, simulations indicate that a 5–10-fold throughput improvement may be achieved using the pointwise acquisition mode.

## Introduction

Mass spectrometry imaging (MSI) is a label-free molecular imaging technique, which enables mapping of multiple classes of molecules in biological tissues.^1−6^ MSI technologies usually use a laser beam, cluster beam, or small liquid volume to desorb and ionize analytes. Subsequently, a mixture of ionized molecules is analyzed by using a mass spectrometer. Matrix-assisted laser desorption/ionization (MALDI) is the most popular ionization technique for MSI.^7^ Other widely used ionization methods include secondary ion mass spectrometry (SIMS),^8^ desorption electrospray ionization (DESI),^9^ and nanospray desorption electrospray ionization (nano-DESI).^10^ After four decades of development in both sampling and acquisition, MSI enables imaging of hundreds of molecules at a cellular scale with high sensitivity and specificity.

Current developments in this field focus on enhancing the spatial resolution, throughput, and molecular coverage.^11−13^ For example, with a specially focused laser beam and postionization, the pixel size has been reduced to ∼1 μm.^14−16^ Meanwhile, the spatial resolution of liquid extraction-based imaging has been improved from ∼100 μm to better than 10 μm.^17,18^ Several strategies have been used to improve the molecular coverage. For example, ion mobility spectrometry has been coupled with MSI to separate ions based on their structures and charge states, which increases the depth of coverage and enables the differentiation of isobaric ions in the gas phase.^19−21^ In addition, isomer-selective imaging of unsaturated lipids has been achieved by combining chemical derivatization with tandem mass spectrometry of the products.^22−26^ Although these developments substantially enhance the analytical performance of MSI, they often increase the total acquisition time by either sampling more positions to increase the spatial resolution or increasing the per-pixel acquisition time for obtaining a broader molecular coverage.

The relatively low experimental throughput of MSI is a major obstacle for several important applications. For MSI to replace traditional hematoxylin and eosin (H&E) microscopy in intraoperative tissue analysis, the experiment and data analysis must be finished in less than 30 min.^27−29^ 3D MSI is another application which is limited by the experimental throughput. 3D ion images create depictions of molecular distributions in physical volumes, which is advantageous for visualizing complex interrelationships between anatomical structures.^30−32^ 3D MSI images are usually generated through serial sectioning of a tissue, followed by 2D imaging and co-registration of the individual sections. 3D imaging experiments often image dozens of sections for the same tissue, which is only practical with high throughput.

Several strategies have been developed to improve the throughput of MSI experiments. High-throughput MALDI MSI with an acquisition rate of 50 pixels/s was performed using a Nd:YLF solid state laser with high repetition rate in a continuous raster scan mode.^33,34^ Equipped with a galvanometer-based optical scanner, a time-of-flight (TOF) MALDI instrument has achieved an acquisition rate of 100 pixels/s in a laser scanning mode.^35^ A parallel ion accumulation and detection approach has been implemented in Fourier-transform ion cyclotron resonance (FT-ICR) MSI to significantly shorten data acquisition time.^36^ Additional computational approaches have been developed. For example, a subspace modeling approach has been used to accelerate FT-ICR MSI by reconstructing high-resolution mass spectral data from short transients.^37^ A follow-up study coupled the subspace modeling method with compressed sensing to reconstruct MSI images from a sparse set of randomly selected locations, reducing the total number of pixels to be sampled and thereby, the acquisition time.^38^ However, current compressed sensing methods based on stochastic process are computationally expensive, which limits their applicability to on-the-fly implementations.

A recently developed deep learning approach for dynamic sparse sampling (DLADS)^39^ is compatible with the hardware of MSI systems and provides an additional improvement of the experimental throughput. DLADS uses an iterative prediction to direct data acquisition to molecularly informative locations. MSI data acquisition time can be reduced by reconstructing ion images, with high fidelity, using only a small portion of the total sample pixels. The DLADS algorithm stems from the supervised learning approach for dynamic sampling (SLADS), which has been successfully applied to scanning electron microscopy,^40^ X-ray diffraction mapping,^41^ and confocal Raman microscopy.^42^ In DLADS, the algorithm was redesigned for compatibility with the MSI data acquisition using a trained U-Net convolutional neural network (CNN) model to utilize interpixel spatial relationships.^39^ DLADS also uses multiple *m*/*z* channels in its decision-making process to consider the molecular heterogeneity of the sample.

This work evaluates the performance of the DLADS algorithm both in its pointwise and segment linewise acquisition modes. Each pixel is sampled independently in the pointwise mode, while in the linewise mode a group of sampling positions along one line is selected in each iteration.^39^ Pointwise DLADS is suitable for laser-beam-based MSI techniques, such as MALDI, in which each sampling event is independent.^35,43^ In contrast, this study uses nano-DESI, where imaging data are acquired as line scans.^44^ To support this mode of data acquisition, linewise DLADS mode was developed.^39^ Herein, the performance of DLADS in both acquisition modes is compared by simulating with fully acquired nano-DESI MSI data of a mouse kidney tissue. The hardware and software integration required to incorporate DLADS into the experimental workflow is described culminating in the first experimental implementation of DLADS on a commercial mass spectrometer equipped with a nano-DESI MSI platform. Linewise DLADS demonstrates a 2.3-fold improvement in the experimental throughput, while generating high-quality reconstructed molecular images. Meanwhile, simulations indicate that a 5–10-fold improvement in throughput may be achieved in the pointwise data acquisition mode. This approach is complementary to other technology development efforts in high-throughput MSI and may be used in combination with other approaches to further expand the range of MSI applications in biological research.

## Theoretical Methods

This section briefly describes the theoretical framework of the DLADS iterative approach for dynamically identifying and sampling a set of sparse tissue locations, leading to high fidelity molecular image reconstructions. Prior to each sampling iteration, DLADS uses a CNN model to generate estimated reduction in distortion (ERD) values for as-of-yet unmeasured locations. This entropy metric acts as a measure of how molecularly informative different locations may be with regard to the final reconstructions. The details of the DLADS algorithm have been previously reported.^39^ The DLADS program incorporates model training, simulation, reconstruction, and experimental implementation functionalities. The source code for DLADS has been made available under the GNU General Public License v3.0 at https://github.com/Yatagarasu50469/SLADS.

Consider an MSI experiment designed to sample a multidimensional tissue *X* with *N* × *M* pixels. There exists a set of *S* locations, with ion intensities in multiple *m*/*z* bins denoted by *X*^(*S*)^. The remaining unsampled locations are denoted as *T*, with the corresponding ion intensities as *X*^(*T*)^. *U* specifies the set of to-be-sampled pixel locations, with the corresponding ion intensities defined with *X*^(*U*)^. Information at unsampled locations can be estimated using a weighted mean interpolation function (*X̂*^(·)^). Image reconstructions comprise *m*/*z*-specific ion intensity values from the sampled locations and unsampled locations: [*X*^(*S*)^,*X̂*^(*T*)^)]. Required for model training and simulation evaluation, the actual reduction in distortion (RD) is described by the difference between the reconstructions with and without a measurement, as shown in eq 1, where *R* denotes the reduction and *D*(·,·) denotes the absolute difference between two images. Subsequent Gaussian filters are applied on a pixel-by-pixel basis, in order to account for regional effects. The selection of the most informative sampling locations during active sampling relies on the maximization of ERD. Since *X* cannot be fully known during the experiment, DLADS uses a U-Net CNN model (Figure S1) represented by *g*^*w*^(·) to compute an ERD map, leveraging interpixel spatial relationships, as shown in eq 2. The model input comprises three *N* × *M* arrays, where the reconstruction values (*X̂*^(*T*)^), measured values *X*^(*S*)^, and measurement locations (*X*^(*S*)^ > 0) are mapped to their 2D locations. The pointwise mode selects a singular location with the highest ERD value in each iteration as a default producing visualizations for every 1% sampled. The segment linewise mode first selects the line with maximal sum ERD and then applies Otsu thresholding to obtain a final set of measurement locations. ERD cannot be produced without information; therefore, a user-specified number of pixels are initially sampled. In the pointwise mode, 1% of randomly sampled pixels were selected in the assigned imaging region for the DLADS initialization. Meanwhile, the initialization in the linewise mode was performed by sampling of three complete lines at 25%, 50%, and 70% of the sample height. After the initial measurements the DLADS model imports the corresponding ion intensities, performs image reconstruction for each *m/z*, generates an ERD map and determines subsequent sampling location(s). This process is iteratively performed until a stopping condition (e.g., % pixels sampled) is satisfied.
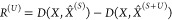
1
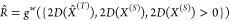
2The model (*g*^*w*^(·)) weights are trained with fully measured MSI data, with *dm*/*z* channels, randomly resampled from 1 to 30% at 1% intervals, to produce sets of expected inputs: sparse measurement values and reconstructions for all chosen *m*/*z* channels; and outputs: ground-truth RD maps. Layer weights (*w*) are optimized using Nadam optimizer and mean absolute error loss between the ground-truth RD (*R*) and ERD values (*R̂*), as shown in eq 3. Multiple ion channels are used in the DLADS training, testing, and implementation to obtain accurate molecular distributions for different types of molecules observed experimentally. Specifically, several representative *m*/*z* images with distinct molecular distributions can be selected using a self-supervised molecular colocalization clustering approach.^45^ Pixels with the highest mean ERD values from all m/z channels are then preferentially sampled in acquisition steps. For the simulation study, the DLADS model was trained with RD formed independently for each *m/z* channel, where during testing and implementation, the ERD matrices of each *m*/*z* channel were averaged together using eq 4.
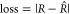
3
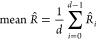
4The DLADS models were trained using nano-DESI MSI data obtained for mouse uterine tissue, as described in a previous study.^39^ Two of the samples were reserved as a validation set for early termination of model training.

To quantitatively evaluate the image reconstruction fidelity of DLADS in the simulation, the reconstructed images and fully sampled images were compared using a standard image metric: peak signal-to-noise ratio (PSNR) in units of dB, calculated using eq 5,
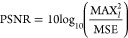
5where MAX_*I*_ is the maximum possible pixel value of the image and MSE is the mean squared error. The calculation of MSE for two images *I* and *K* with *N* × *M* dimensions is shown in eq 6.
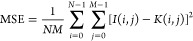
6PSNR manages high dynamic ranges and approximates human perception of image reconstruction by weighing the MSE against the MAX_I_. A higher value of PSNR indicates a better overall quality of image reconstruction. Therefore, PSNR values were used to evaluate the quality of image reconstructions in DLADS simulations.

## Experimental Methods

### Tissue Section Preparation

Fresh frozen C57BL/6 mouse kidney tissues used in this study were purchased from BioIVT (Westbury, NY, USA). Kidney tissues were sectioned using a CM1850 Cryostat (Leica Microsystems, Wetzlar, DE, USA) and thaw-mounted onto glass microscope slides (Tek-Select Gold Series Microscope Slides, Clear Glass, Positive Charged, IMEB, inc., CA, USA). Section thickness was controlled at 12 μm.

### Nano-DESI MSI

**Figure 1:**
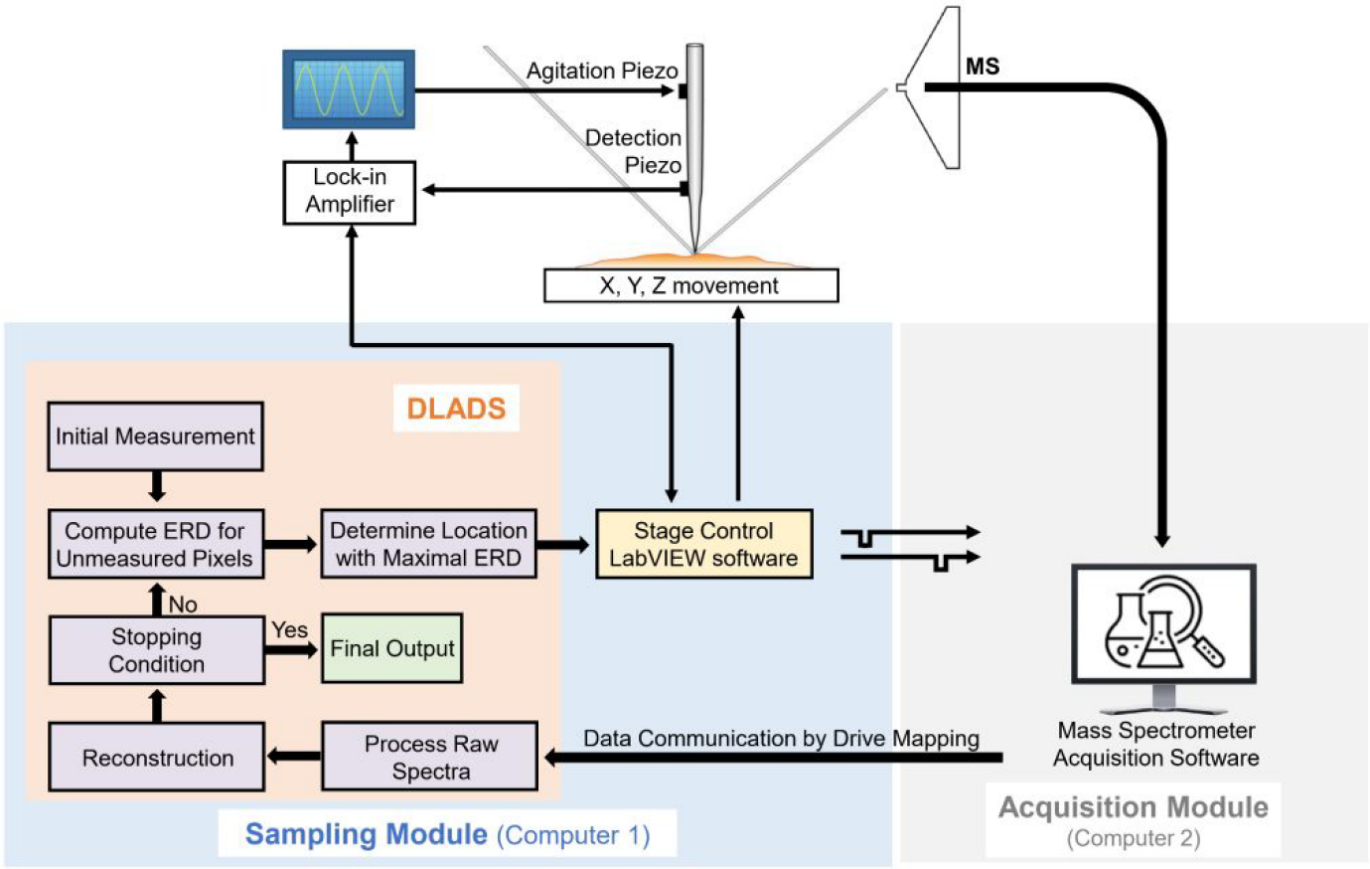
Overview of the dynamic sparse sampling nano-DESI MSI system coupled with DLADS. Black arrows indicate the data flow for DLADS computation, stage control, and data acquisition.

Mouse kidney tissue sections were analyzed using an Agilent 6560 IM-QTOF (Agilent Technologies, Santa Clara, CA, USA) equipped with a custom designed nano-DESI source,^17^ as shown in Figure 1. The nano-DESI probe is composed of two fused silica capillaries (OD 150 μm × ID 50 μm) that transfer the extraction solvent to and from the sample. Analytes are extracted into a liquid bridge formed between the two capillaries and sample, transferred to a mass spectrometer (MS) inlet, and ionized using nanospray ionization. A third pulled capillary serves as a shear force probe to maintain a constant distance between the capillary probe and tissue surface.^46^ More specifically, two piezoelectric ceramic plates (3.8 MHz, Steiner & Martins, Inc., Doral, FL, USA) are attached to the shear force probe for detecting the amplitude of shear force vibration with a lock-in amplifier (Stanford Research Systems, Sunnyvale, CA, USA). The z-position of the sample stage (Zaber Technologies Inc., Vancouver, BC, CA, USA) is automatically adjusted to maintain a constant amplitude of the shear force vibration at a selected resonance frequency, which is sensitive to the sample surface. This feedback control system is controlled by a custom designed LabVIEW software. MS data were acquired in positive ionization mode using 9:1 MeOH/H_2_O (v/v) as a solvent, which was infused using a LEGATO 100 syringe pump (KD Scientific Inc., Holliston, MA, USA) at 0.5 μL/min. Ionization was achieved by applying a high voltage of +4 kV to the syringe needle. Nano-DESI MSI data were acquired in lines by scanning the sample stage in one direction at a scan rate of 40 μm/s and stepping by 150 μm between the lines. This method was used to acquire MSI data for a mouse kidney tissue section without sparse sampling, referred to as “fully measured” data later in the text. The fully acquired data were used as ground-truth references in the DLADS simulation and implementation studies.

### Integration of the Linewise DLADS with Nano-DESI Platform

DLADS integration with nano-DESI MSI, shown in Figure 1, combines sampling and acquisition modules, which are controlled by two computers. In the acquisition module, Agilent’s MassHunter software controls the IM-QTOF system. Furthermore, MassHunter accepts contact closure signals from the stage control LabVIEW software, in order to start and end the acquisition. It also saves MS data to the DLADS program folder on a mapped network drive. The DLADS program, in the sampling module, interfaces with the MS acquisition and stage control software with a polling operation to automate dynamic sparse sampling. During a sampling iteration, once DLADS loads the latest MS data, it updates and reconstructs ion images for user-specified channels. The pretrained CNN model predicts ERD for the unmeasured locations, using sampled locations, measured ion images, and reconstructed ion images for inputs, as shown in Figure S1. After the ERD map is obtained, a set of line-bounded locations with maximal ERD values are selected for the next acquisition. Once the stage control software polls the latest line sampling locations, it directs the sample stage to the starting location of the next line. Next, the nano-DESI probe lands on the tissue surface with the shear force feedback control system.^47^ The stage control software then sends a contact closure to Agilent’s IM-QTOF-MS to start acquisition, using a digital to analog converter (USB-6009, National Instruments Crop., Austin, TX, USA). After the line scan finishes, the stage control software sends another contact closure to end the MS data acquisition, and the sampling moves into the next iteration until a stopping condition is satisfied. The source code for the stage control LabVIEW software is available at https://github.com/LabLaskin/nano-DESI-stage-dynamic-sampling. The DLADS and LabVIEW software ran on a computer equipped with an Intel i5-8500 CPU and 8 GB RAM.

## Results and Discussion

### Simulation Results

**Figure 2:**
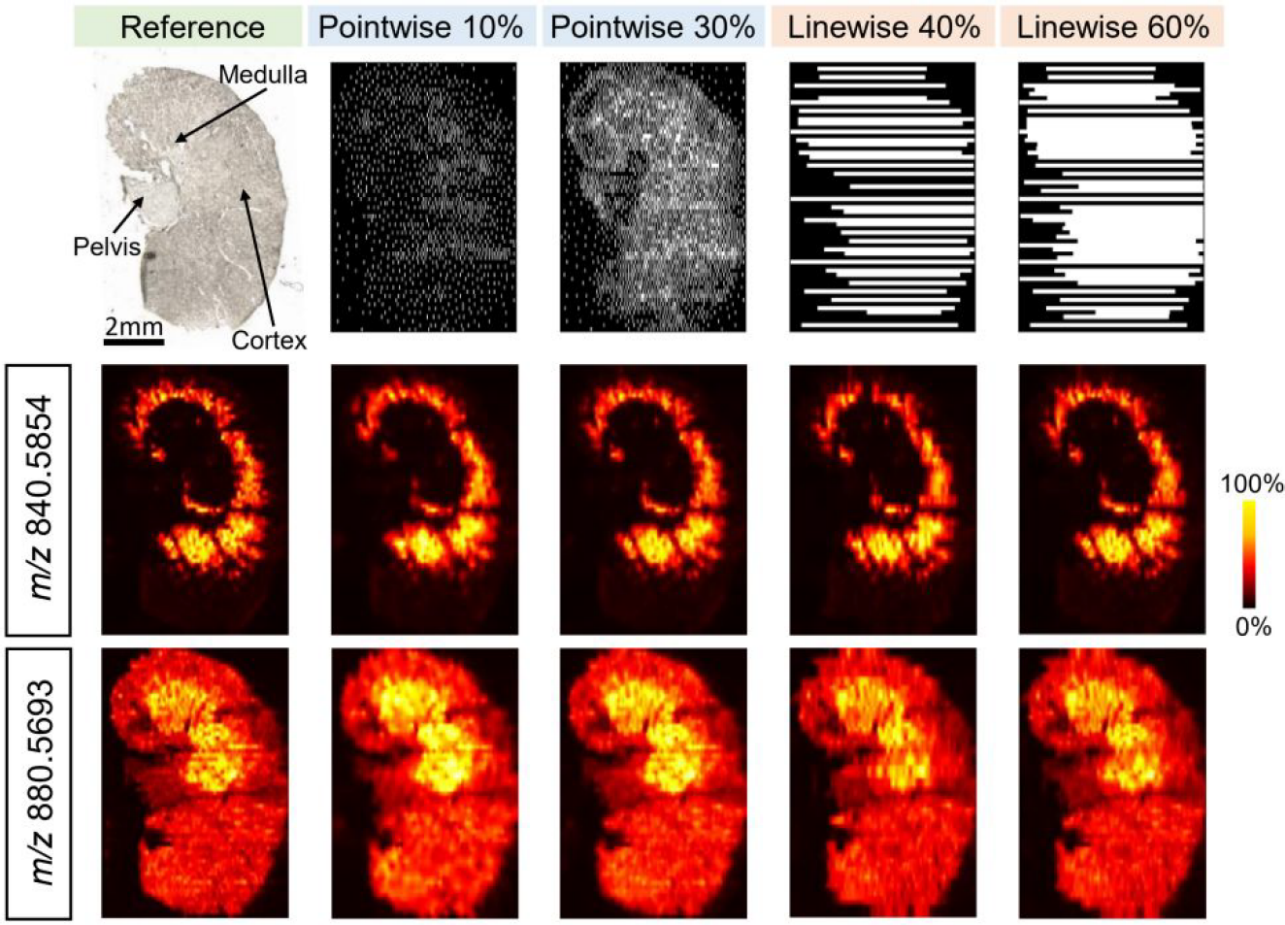
Simulated DLADS dynamic sampling and reconstruction in the pointwise and linewise modes, using a fully measured mouse kidney tissue MSI data. An optical image and ground-truth ion images of *m*/*z* 840.5854 and *m*/*z* 880.5693 are shown in the first column. DLADS sampling locations and reconstructed ion images at different simulation conditions are shown in columns 2–5. The results are shown for 10% and 30% sampling in the pointwise mode and 40% and 60% sampling in the linewise mode.

Simulations were used to evaluate the performance of DLADS. The simulations used nano-DESI MSI data of a mouse kidney tissue acquired without sparse sampling on the Agilent IM-QTOF MS instrument as a ground-truth reference. A fully measured MSI data refers to data acquired by sampling all the predefined locations on the sample. For the linewise acquisition, this involves acquiring line scans across a tissue section with a constant user-defined step between the lines. The fully measured data was digitally resampled to simulate dynamic sparse sampling, allowing for direct comparison of the reconstructed and ground-truth images. The simulations utilized both pointwise and linewise modes by monitoring six *m*/*z* channels shown in Figure S2 to generate representative results shown in Figure 2. An optical image and two ground-truth ion images of the mouse kidney section are shown in the first column. One ion at *m*/*z* 840.5854 is distributed at the inner cortex region. Another ion at *m*/*z* 880.5693 shows a different localization. It is distributed throughout the whole tissue and is enhanced in the medulla region. The fine distinctions between the patterns of the two ion images help qualitatively evaluate the DLADS sampling and reconstruction performance.

The first row of the DLADS simulation results (columns 2–5) shows the sampling locations, where the other two rows contain the reconstructed ion images for *m*/*z* 840.5854 and 880.5693. Reconstructed ion images of other *m*/*z* values are shown in Figure S3. Reconstructions with 10% sampling in the pointwise mode resembles ion distributions in available reference images. When the sampling density increases to 30%, locations in both the inner cortex and medulla regions are preferentially sampled. As a result, some structural details, such as the distribution of *m*/*z* 840.5854 in the tubules within the inner cortex and *m*/*z* 880.5693 distribution in the medulla, are reconstructed with higher fidelity. The distribution of the sampling locations in the pointwise mode demonstrates that molecularly informative locations are effectively identified by the DLADS algorithm. The linewise sampling mode imposes a spatial constraint that pixels along one line must be sampled in one iteration, thereby only allowing sparse sampling in one dimension. Image reconstruction using 40% pixels sampled in the linewise mode provides a result similar to that obtained using 10% sampling in the pointwise mode. Using 60% sampling in the linewise mode, fine details including the tubule edges in the distribution of *m*/*z* 880.5693 are well-resolved, as annotated in Figure S4. The results of dynamic sparse sampling in the linewise mode were also compared, with the images obtained by uniformly sampling 40% of the pixels as shown in Figure S5. Better resolved spatial features in the tubule and medulla regions obtained using sparse sampling further confirm that the DLADS approach effectively guides acquistion to molecularly informative locations, providing better-quality data than uniform under sampling. For both sampling densities, on-tissue pixels are preferentially sampled, while only a small number of pixels, primarily obtained during initialization, are sampled outside of the tissue where there are no substantial chemical gradients. The effective sampling and reconstruction in both modes demonstrate the generalization capability of the DLADS algorithm.

**Figure 3:**
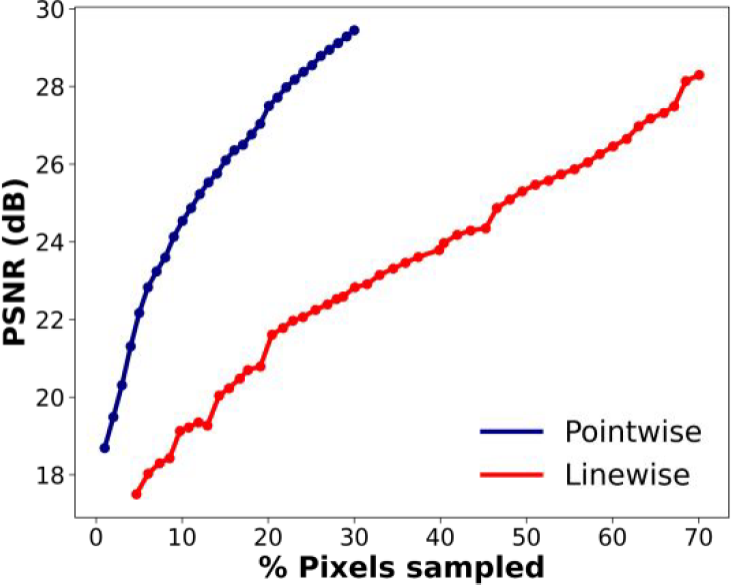
PSNR as a function of sampling density in both the pointwise and linewise modes.

Quantitative evaluation of the reconstructed image quality, obtained using DLADS sampling, is shown in Figure 3. In this simulation, DLADS used six different *m*/*z* channels, which represent the molecular heterogeneity of the tissue sample. For each DLADS sampling iteration, reconstructed ion images and their calculated PSNR values with respect to ground-truth ion images were generated, as described in the experimental section. The average PSNR values are plotted as a function of sampling density in Figure 3 for both the pointwise and linewise modes. Across all sampling densities, the PSNR values obtained for the pointwise mode are higher than those obtained for the linewise mode. These results demonstrate that pointwise sampling in DLADS provides high-quality reconstructed ion images using a smaller sampling density compared to linewise operation. For example, the PSNR value of 24 dB was obtained using 10% and 40% sampling densities in the pointwise and linewise acquisition modes, respectively, while at 30% sampling density the values were 29.1 and 22.8 dB.

Also evaluated was how the selection of *m*/*z* channels used in DLADS affects the selection of sampling locations. In the original simulation, six *m*/*z* channels (Figure S2) were used, with distinct spatial distributions in the mouse kidney tissue sample. To illustrate the importance of *m*/*z* channel selection for DLADS sampling and reconstruction, an additional simulation was performed with fewer *m*/*z* channels, as shown in Figure S6. In this simulation, sampling was guided by ion images of only two *m*/*z* channels (741.5259 and 845.5273, shown in panel A) enhanced in the cortex region of the mouse kidney tissue. With this guidance, the pointwise sampling locations and reconstructed images of *m*/*z* 840.5854 and 880.5693 are shown in panel B, at 10% and 30% sampling densities, to compare with the results shown in Figure 2. It was observed that the locations in the cortex were preferentially sampled in this DLADS simulation, indicating that other locations, in which the two monitored *m*/*z* channels have lower signals, were regarded as less informative regions. As a result, the ion image of *m*/*z* 840.5854 enhanced in the inner cortex was constructed with high fidelity. Meanwhile, the chemical gradient of *m*/*z* 880.5693 in the medulla region (highlighted by arrows in Figure S6B) is not well reproduced in the reconstructed image shown in Figure S6B, as compared to the result shown in Figure 2. Quantitative analysis using the PSNR metric, shown in Figure S6C, also confirms these qualitative observations. These results indicate that DLADS reconstruction may be biased by the selection of *m*/*z* channels. However, a robust reconstruction of ion images has been achieved using a combination of representative *m*/*z* channels in DLADS calculations, as shown in Figure S3. It may be noted that a careful selection of *m*/*z* channels may be used for targeted applications, such as clinical diagnostics and drug screening.

**Figure 4:**
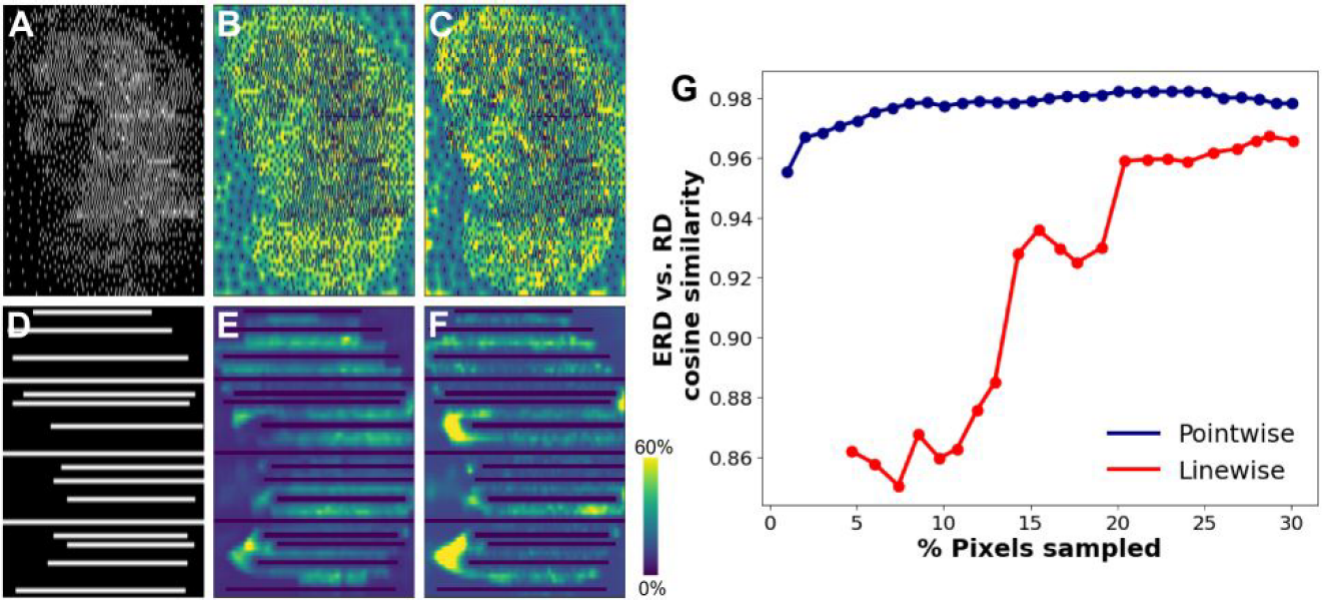
Evaluation of the ERD in both the pointwise (A–C) and linewise (D–F) acquisition modes. Sampling locations (A, D), ERD matrices (B, E), and RD matrices (C, F) obtained using a 20% sampling density in the pointwise and linewise acquisition modes, respectively. (G) Cosine similarity scores between the predicted ERD and RD values as a function of the sampling density obtained for both acquisition modes.

Examination of the ERD spatial distributions provides insight into DLADS performance for the pointwise and linewise modes. Since DLADS prioritizes acquiring locations with higher ERD values, a robust estimation of the RD values is critical to the effectiveness of the dynamic sampling. Figure 4 visualizes the sampled locations (panels A and D), along with the ERD (panels B and E), and RD (panels C and F) matrices, obtained for 20% sampling density in both pointwise (panels A–C) and linewise (panels D–F) modes. The ERD and RD values are shown as heat maps, where bright yellow indicates high values, while dark blue indicates low values. Intensities were limited for visualization to the range of 0–60% to eliminate spikes in their distributions. The ERD matrix in the pointwise mode (Figure 4B) resembles the ground-truth RD matrix (Figure 4C), which indicates that the CNN model accurately predicts molecularly informative sampling locations. In contrast, noticeable differences can be seen between the ERD (Figure 4E) and RD (Figure 4F) matrices in the linewise mode. The ground-truth RD values in unsampled lines are more heterogeneous, reflecting the nonuniform distributions of molecules in the mouse kidney tissue, as shown in Figures 2 and S3. These observations are further confirmed by the cosine similarity score trend between the ERD and RD values, plotted as a function of the sampling density for both modes in Figure 4G. For a wide range of sampling densities, the similarity scores in the pointwise mode are greater than 0.96, again indicating the ERD prediction is highly effective. The similarity scores in the linewise mode increase with the number of sampled pixels. However, all the scores calculated for the linewise mode are lower than those obtained for the pointwise mode. Overall, the evaluation of the ERD values indicates that the trained CNN model effectively estimates the RD values for selecting molecularly informative sampling locations, while the line-bounded geometry constraint reduces the performance.

### Implementation Results

**Figure 5:**
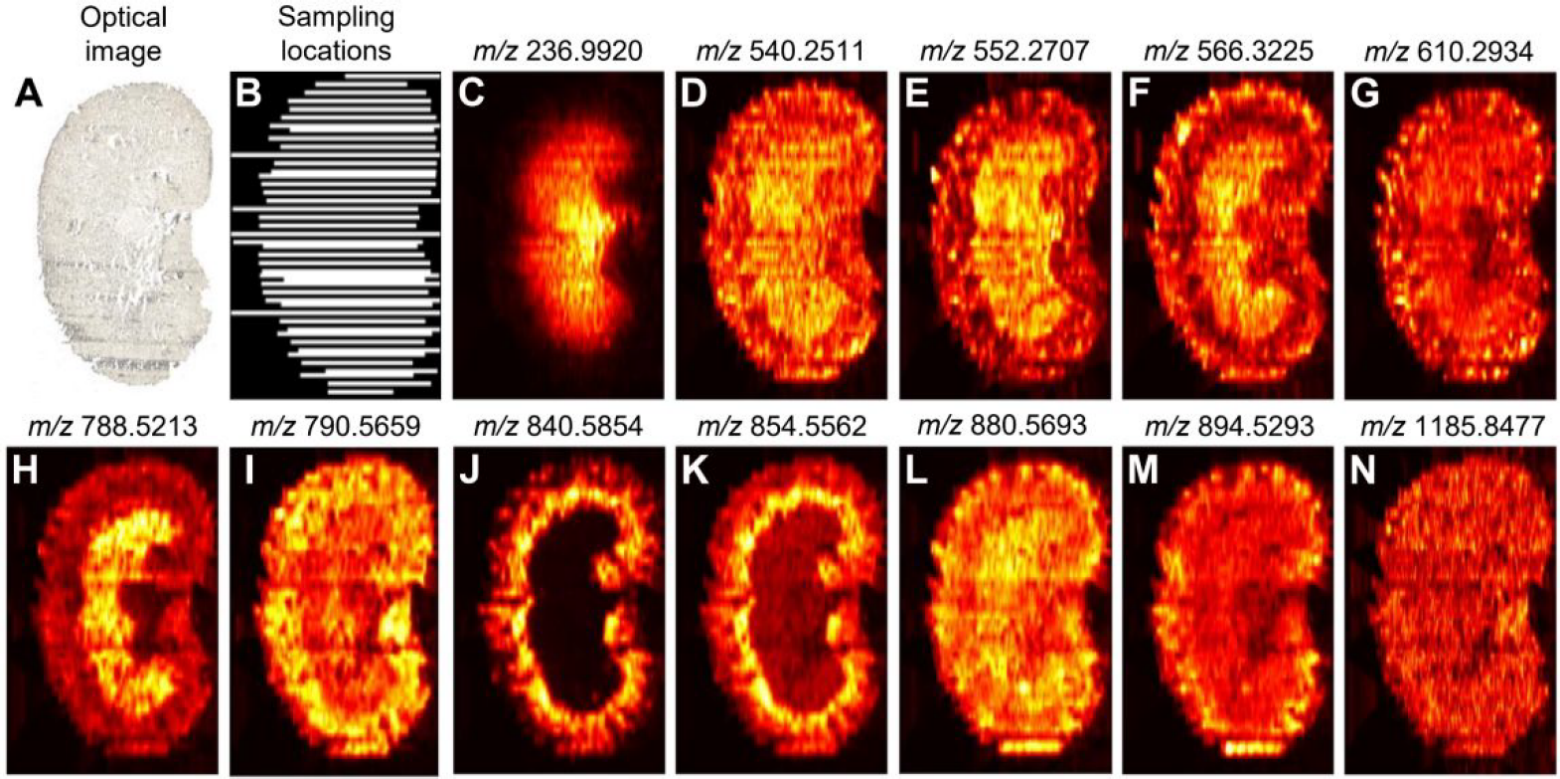
Experimental implementation of DLADS using a mouse kidney tissue section. (A) Optical image. (B) Line-bounded sparse sampling locations. (C–N) Reconstructed ion images.

This work demonstrates the feasibility of dynamic sparse sampling in the linewise mode by implementing DLADS on a nano-DESI imaging platform. In proof-of-concept experiments, imaging of a mouse kidney tissue section was performed using the same six *m*/*z* channels as those used in simulations (Figure S2) and a stopping condition of reaching a 40% sampling density. The results of this experiment are summarized in Figure 5. An optical image of the tissue section is shown in Figure 5A, and the experimentally sampled locations, dynamically selected by DLADS, are shown in Figure 5B. With 40% of sampled pixels, the reconstructed ion images of different *m*/*z* values provide distinct molecular distributions with high fidelity (Figure 5C–N). The reconstructions were compared with fully measured images, shown in Figure S3, acquired using another kidney tissue section from the same mouse model. The consistency of molecular distribution in kidney subregions, obtained with and without sparse sampling, further confirms that the spatial molecular information is well-preserved in the compressed DLADS MSI data. In addition, the experimental throughput of DLADS was evaluated according to different experimental configurations, summarized in Table 1. A regular nano-DESI MSI experiment acquires imaging data by scanning the sample surface in a line-by-line manner. The experiment for each line can be divided into (a) scan preparation, in which MS software (Agilent MassHunter) becomes ready for acquisition and the stage system lands the nano-DESI probe onto a sample surface, and (b) MS acquisition, where the nano-DESI probe scans a line on the tissue using a constant scan rate. The scan rate is determined by the desired spatial resolution and acquisition rate of a mass spectrometer. Referencing Table 1, the average scan preparation time was only about 9 s per line. DLADS adds a computational step to the regular workflow that involves reading of the raw MS data, computing the ERD using a trained CNN model, and assigning locations for the next sampling. With an Intel i5-8500 CPU, the average DLADS computation time was just 5 s per line. For the sample area of 11.7 × 7.5 mm, the total DLADS acquisition time took 111 min. Meanwhile, the acquisition time for a full image without sparse sampling is estimated to be 259 min. Linewise DLADS therefore provides a 2.3-fold improvement in the nano-DESI experimental throughput with a small overhead that can be further reduced with updated computational hardware. The experimental throughput of the pointwise acquisition may be improved by a factor of 5–10 using DLADS.

**Table 1:** Experimental Properties for the Linewise DLADS Implementation Using a Mouse Kidney Tissue Section

parameters	values
sample dimension (mm)	11.7 × 7.5
scan rate (μm/s)	40
scheduled lines	79
sampled lines	44
MS acquisition time (min)	100
scan preparation time (min)	7
DLADS computation time (min)	4
estimated full imaging time (min)	259

## Conclusions

A high-throughput nano-DESI MSI system was developed by coupling it with dynamic sparse sampling, performed using DLADS. This work describes the hardware and software integration for the experimental implementation of DLADS. Using multiple *m*/*z* channels, the trained DLADS U-Net CNN model effectively predicts molecularly informative sampling locations. This computation-guided sampling dynamically adjusts throughout an MSI experiment, significantly reducing the number of sampling locations required for reconstructing high-fidelity molecular images. Although the DLADS CNN model was trained using MSI data for mouse uterine tissue acquired on a Thermo Orbitrap instrument, both the simulations and experimental implementation reported in this study were performed using mouse kidney tissue sections imaged on an Agilent IM-QTOF instrument. The results demonstrate the robustness of the DLADS algorithm and its generalization power, making it applicable to different tissue types and different mass spectrometry platforms. Herein, simulations were used to evaluate two distinct acquisition modes: a pointwise mode applicable to MALDI MSI experiments and a linewise acquisition mode typical of nano-DESI MSI experiments. Simulations on a mouse kidney tissue demonstrate that pointwise DLADS achieves a 24.5 dB reconstruction with only 10% sampled pixels, indicating a 10-fold improvement in the throughput of MSI experiments. The linewise DLADS has been implemented on a nano-DESI MSI system and demonstrated by imaging mouse kidney tissue sections. Similar to the simulation results, a 2.3-fold improvement in throughput has been acheived experimentally for the linewise acquisition, where the DLADS performance is restricted by the line-bounded geometry constraint. The high-throughput DLADS MSI imaging platform is compatible with different types of mass spectrometers and ionization techniques. Dynamic sparse sampling is a promising approach for the development of robust high-throughput MSI systems for MSI applications, which require rapid data acquisition, such as intraoperative tissue analysis and 3D imaging. By carefully selecting *m*/*z* channels, a targeted DLADS model may be developed for molecular imaging applications in drug screening and disease diagnostics.

## Supporting Information Available

U-Net model configuration, *m*/*z* channels, and complementary sampling and reconstruction ion images in simulations (PDF)
