# Supplementary material for: High-Throughput Mass Spectrometry Imaging with Dynamic Sparse Sampling
Source: ACS Meas Sci Au. 2022 Aug 15;2(5):466–74. doi: 10.1021/acsmeasuresciau.2c00031 (PMC9585637; doi:10.1021/acsmeasuresciau.2c00031)
Supplement: Supplementary file 1 — tg2c00031_si_001.pdf [file tg2c00031_si_001.pdf]

Supporting Information

High-Throughput Mass Spectrometry Imaging with Dynamic Sparse Sampling

Hang Hu[1], David Helminiak[2], Manxi Yang[1], Daisy Unsihuay[1], Ryan T. Hilger[1], Dong Hye Ye[2], and Julia Laskin[1*]

1. Department of Chemistry, Purdue University, West Lafayette, IN 47907, USA

2. Electrical and Computer Engineering; Marquette University; Milwaukee, WI 53233, USA

Corresponding author: Julia Laskin, Tel: 765-494-5464, Email: jlaskin@purdue.edu

Table of Contents

Figure S1………………………………………………………………………........... S2

Figure S2………………………………………………………………………........... S2

Figure S3…………………………………………………………………………... S3 – S5

Figure S4………………………………………………………………………........... S6

Figure S5………………………………………………………………………........... S6

Figure S6………………………………………………………………………........... S7

Reference………………………………………………………………………........... S7

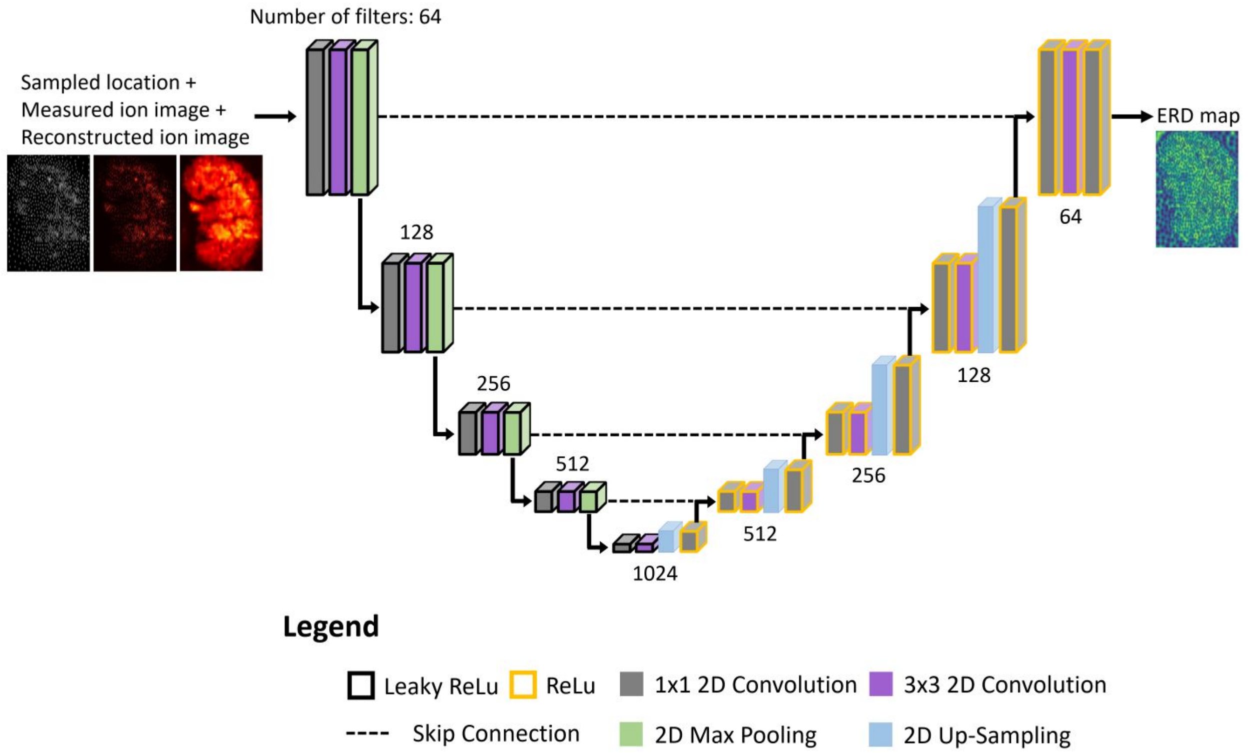

Figure S1. The model structure of U-Net CNN for computing ERD maps.

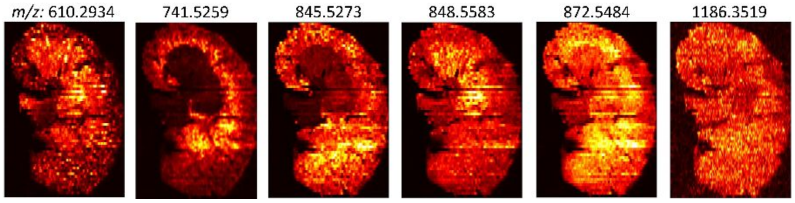

Figure S2. Six *m/z* channels used in DLADS test and implementation for a mouse kidney tissue section.

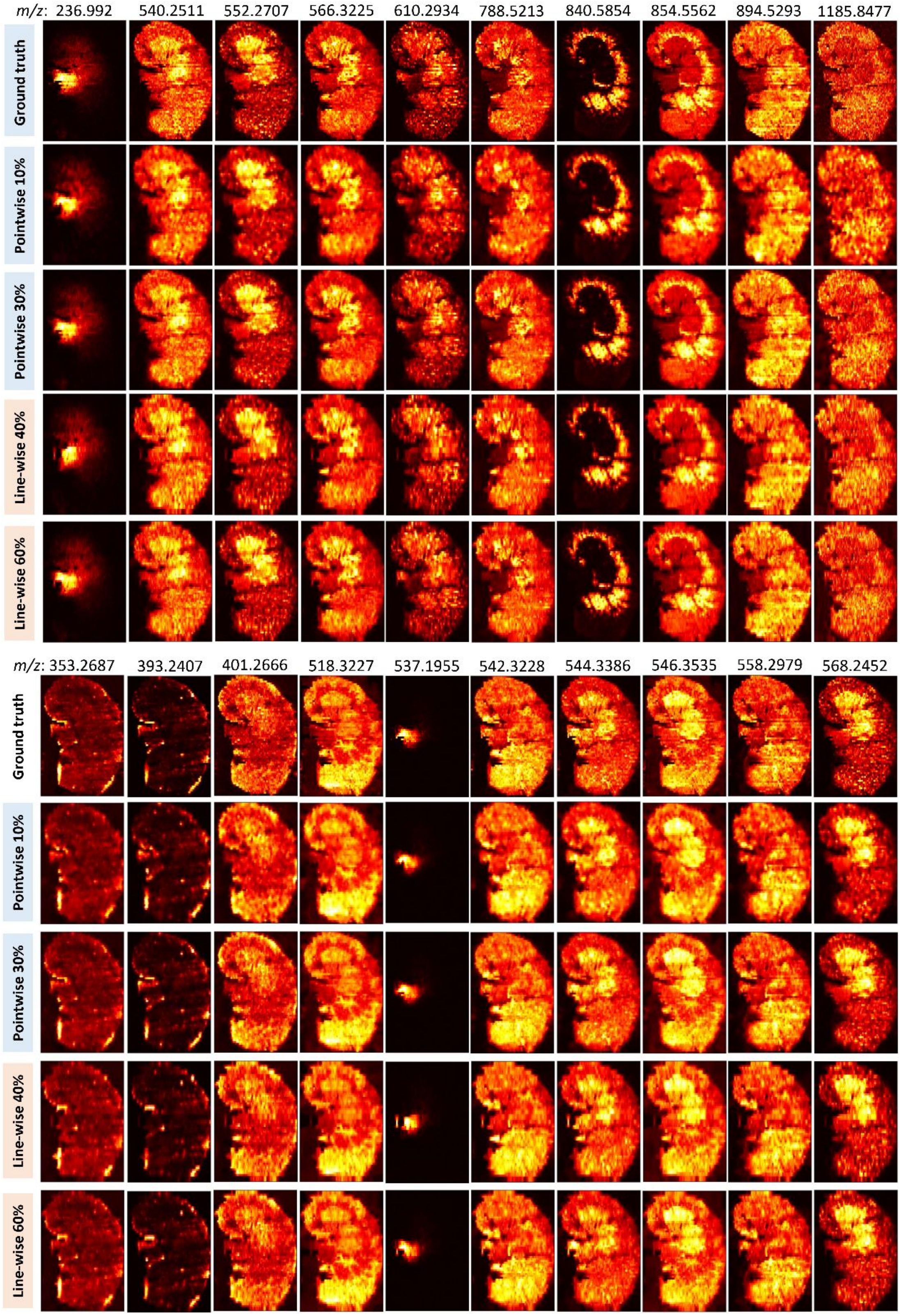
m/z: 236.992
540.2511
552.2707
566.3225
610.2934
788.5213
840.5854
854.5562
894.5293
1185.8477
Ground truth
Pointwise 10%
Pointwise 30%
Line-wise 40%
Line-wise 60%
m/z: 353.2687
393.2407
401.2666
518.3227
537.1955
542.3228
544.3386
546.3535
558.2979
568.2452
Ground truth
Pointwise 10%
Pointwise 30%
Line-wise 40%
Line-wise 60%

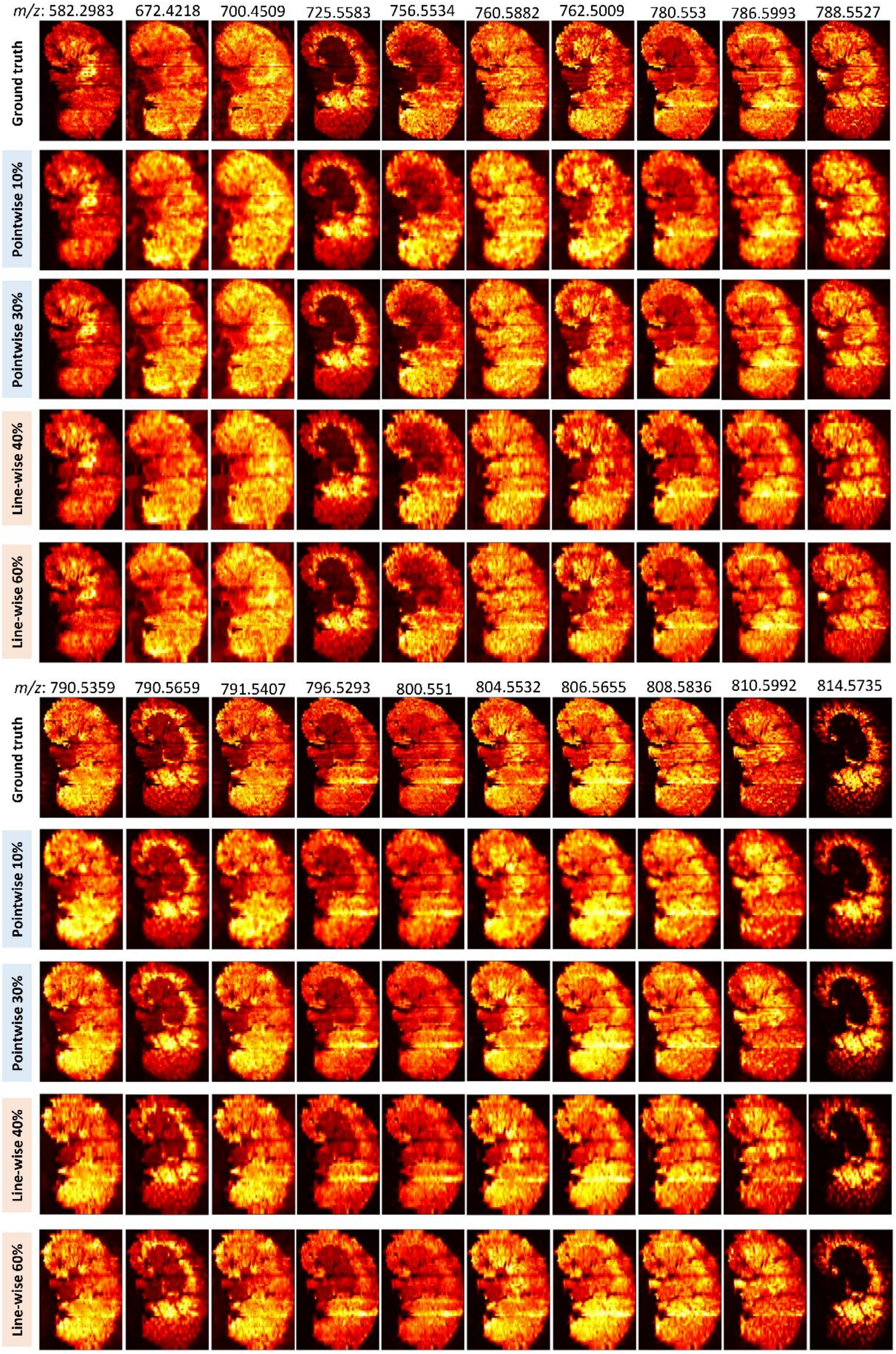
m/z: 582.2983
672.4218
700.4509
725.5583
756.5534
760.5882
762.5009
780.553
786.5993
788.5527
Ground truth
Pointwise 10%
Pointwise 30%
Line-wise 40%
Line-wise 60%
m/z: 790.5359
790.5659
791.5407
796.5293
800.551
804.5532
806.5655
808.5836
810.5992
814.5735
Ground truth
Pointwise 10%
Pointwise 30%
Line-wise 40%
Line-wise 60%

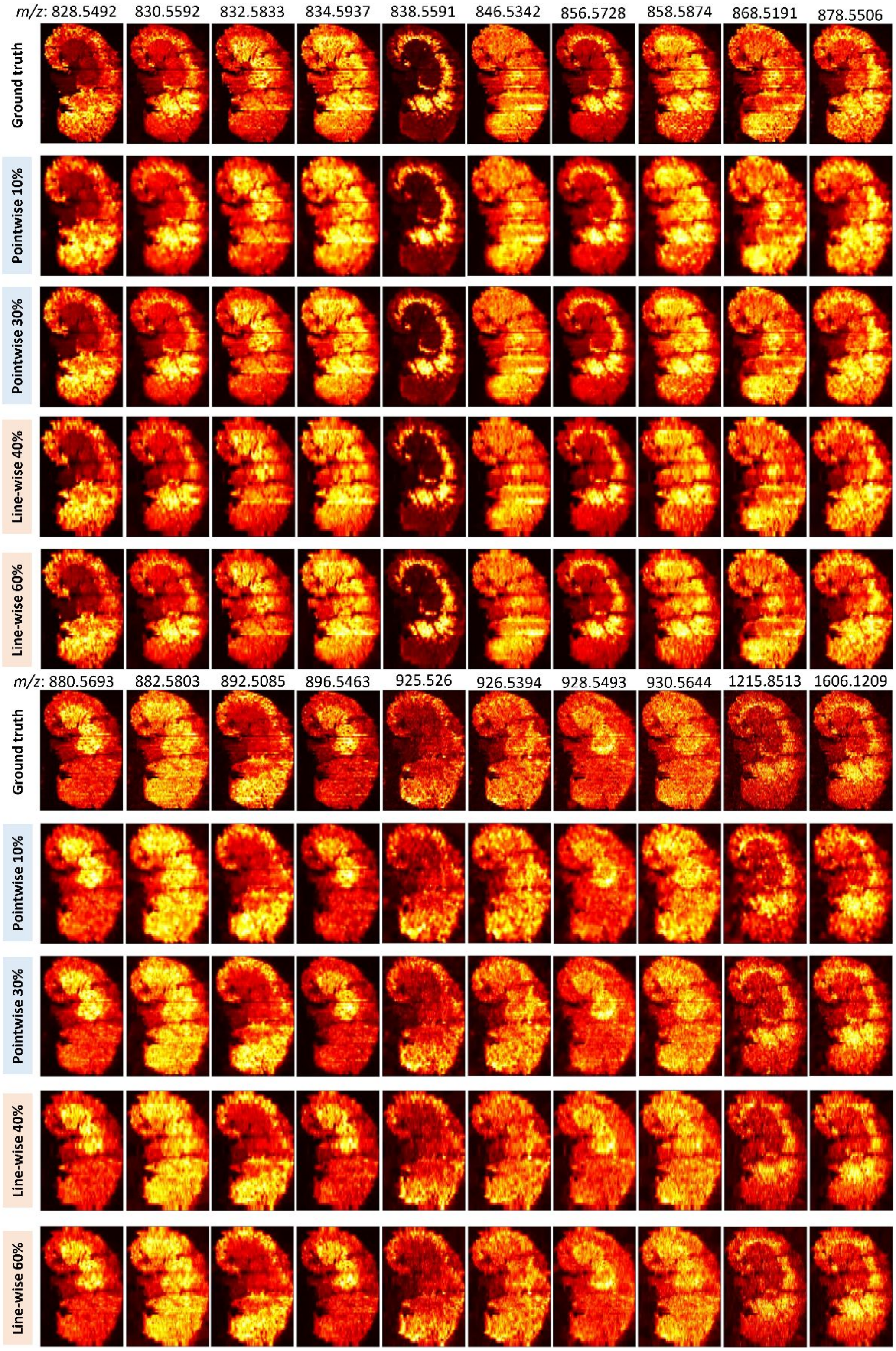

Figure S3. Simulated DLADS dynamic sampling reconstructions from pointwise and line-wise modes for 60 ion images, using fully measured mouse kidney tissue MSI data.

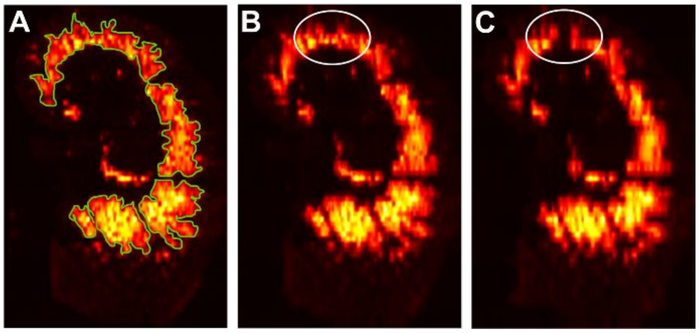

Figure S4. (A) Annotated tubule profile (green) of the mouse kidney inner cortex in the ion image of *m/z* 880.5693. The annotation is made based on previous reports.[1,2] Using 60% sampling in line-wise mode (B), fine details are better resolved than reconstructed ion image with 40% line-wise sampling (C) at tubule edges.

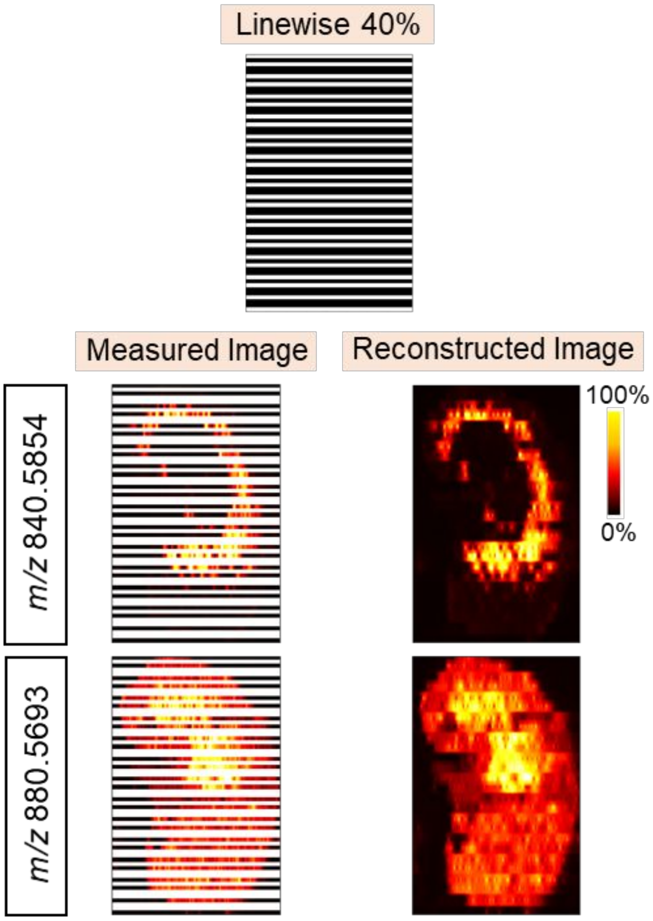

Figure S5. Simulation of uniformly sparse sampling and reconstruction in the line-wise mode using a fully measured mouse kidney tissue MSI data. The sampling locations (white) are shown in the first row. The measured and reconstructed images for *m/z* 840.5854 and *m/z* 880.5693 are shown in the 2-3 rows.

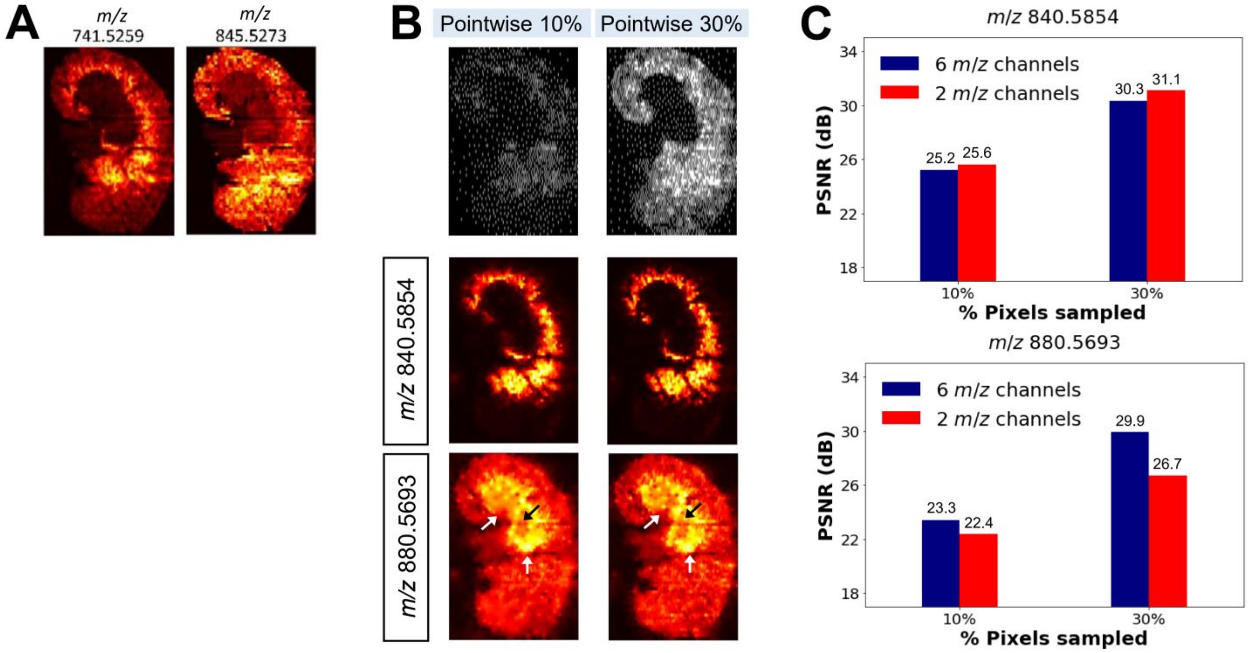

Figure S6. Simulation of DLADS by monitoring two *m/z* channels. (A) Two *m/z* channels used in the DLADS simulation. (B) DLADS sampling locations (first row) and ion image reconstructions (2-3 rows). The white arrows indicate the poorly resolved molecular distributions at kidney medulla. (C) PSNR comparison of reconstruction ion images between Figure 2 and panel B. With more samplings guided in kidney cortex region, the reconstruction of two-channel-monitoring DLADS for *m/z* 840.5854 shows slightly higher fidelity than six-channel-monitoring DLADS. However, the performance for *m/z* 880.5693 deteriorates because of the lack of sampling in the medulla region.

Reference

1. Spraggins, J.M., Djambazova, K.V., Rivera, E.S., Migas, L.G., Neumann, E.K., Fuetterer, A., Suetering, J., Goedecke, N., Ly, A., Van de Plas, R. and Caprioli, R.M. High-Performance Molecular Imaging with MALDI Trapped IonMobility Time-of-Flight (timsTOF) Mass Spectrometry. Anal. Chem. 2019, 91, 14552-14560.

2. Hu, H.; Yin, R.; Brown, H. M.; Laskin, J. Spatial Segmentation of Mass Spectrometry Imaging Data by Combining Multivariate Clustering and Univariate Thresholding. Anal. Chem. 2021, 93, 3477−3485.
